# Impact of glycemic control on circulating endothelial progenitor cells and arterial stiffness in patients with type 2 diabetes mellitus

**DOI:** 10.1186/1475-2840-10-113

**Published:** 2011-12-20

**Authors:** Wen-Sheng Yue, Kui-Kai Lau, Chung-Wah Siu, Mei Wang, Guo-Hui Yan, Kai-Hang Yiu, Hung-Fat Tse

**Affiliations:** 1 Cardiology Division, Department of Medicine, Queen Mary Hospital, The University of Hong Kong, Hong Kong SAR, China; 2 Medical Imaging Key Laboratory of Sichuan Province & Affiliated Hospital of North Sichuan Medical College, Nanchong, China; 3 Neurology Division, Department of Medicine, Queen Mary Hospital, The University of Hong Kong, Hong Kong SAR, China; 4 Research Center of Heart, Brain, Hormone and Healthy Aging, Li Ka Shing Faculty of Medicine, The University of Hong Kong, Hong Kong SAR, China

## Abstract

**Background:**

Patients with type 2 diabetes mellitus (DM) have increased risk of endothelial dysfunction and arterial stiffness. Levels of circulating endothelial progenitor cells (EPCs) are also reduced in hyperglycemic states. However, the relationships between glycemic control, levels of EPCs and arterial stiffness are unknown.

**Methods:**

We measured circulating EPCs and brachial-ankle pulse wave velocity (baPWV) in 234 patients with type 2 DM and compared them with 121 age- and sex-matched controls.

**Results:**

Patients with DM had significantly lower circulating Log CD34/KDR^+ ^and Log CD133/KDR^+ ^EPC counts, and higher Log baPWV compared with controls (all *P < 0.05*). Among those 120/234 (51%) of DM patients with satisfactory glycemic control (defined by Hemoglobin A1c, HbA1c < 6.5%), they had significantly higher circulating Log CD34/KDR^+ ^and Log CD133/KDR^+ ^EPC counts, and lower Log baPWV compared with patients with poor glycemic control (all *P < 0.05)*. The circulating levels of Log CD34/KDR^+ ^EPC (r = -0.46, *P < 0.001*) and Log CD133/KDR^+ ^EPC counts (r = -0.45, *P < 0.001*) were negatively correlated with Log baPWV. Whilst the level of HbA1c positively correlated with Log baPWV (r = 0.20, *P < 0.05*) and negatively correlated with circulating levels of Log CD34/KDR^+ ^EPC (r = -0.40, *P < 0.001*) and Log CD133/KDR^+ ^EPC (r = -0.41, *P < 0.001*). Multivariate analysis revealed that HbA1c, Log CD34/KDR^+ ^and Log CD133/KDR^+ ^EPC counts were independent predictors of Log baPWV (*P < 0.05*).

**Conclusions:**

In patients with type 2 DM, the level of circulating EPCs and arterial stiffness were closely related to their glycemic control. Furthermore, DM patients with satisfactory glycemic control had higher levels of circulating EPCs and were associated with lower arterial stiffness.

## Background

It is well recognized that patients with type 2 diabetes mellitus (DM) have accelerated stiffening of the elastic arteries beyond that explained by normal aging [1]. Such increase in arterial stiffness, as measured by pulse wave velocity (PWV) may represent a useful integrated index of vascular status, and has been identified as an independent predictor for cardiovascular related mortality in patients with DM [2]. In patients with DM, one of the postulated mechanisms of increased arterial stiffness is hyperglycemia-induced depletion of endothelial nitric oxide (NO), subsequently leading to endothelial dysfunction [3]. Dysregulation of the NO system has also been shown to be responsible for the depletion and dysfunction of endothelial progenitor cells (EPCs) in patients with type 2 DM [4]. Interestingly, studies also showed that plasma glucose and hemogloblin A1c (HbA1c) levels inversely correlates with the bioavailability of NO in EPCs [5]. Therefore, we hypothesized that poor glycemic control in patients with type 2 DM is associated with depletion of circulating EPC and thus increased arterial stiffness. The purpose of this study was to investigate the relationships between glycemic control with circulating EPCs and arterial stiffness in patients with type 2 DM.

## Methods

### Study population

Consecutive patients with type 2 DM, as defined by the WHO criteria [6] and treated with stable oral hypoglycemic agents and insulin, and cardiovascular medications for at least 6 months were recruited from the medical outpatient clinic. Patients with poorly controlled DM (HbA1c ≥11%), dilated cardiomyopathy, significant valvular heart disease, chronic atrial fibrillation, New York Heart Association class III/IV heart failure, a history of prior atherothrombotic events (including unstable angina, myocardial infarction, stroke and peripheral vascular disease) or stable angina; creatinine level >220 μmol/L, acute infectious disease, chronic obstructive pulmonary disease, hepatic insufficiency and connective tissue disease were excluded. As a result, a total of 234 patients with type 2 DM were eligible for this study.

The control group was recruited from a local health exhibition and consisted of 121 age- and sex-matched healthy subjects with fasting blood glucose level < 6.1mmol/L and no history of diabetes or cardiovascular diseases. A written informed consent was obtained from each subject and the study was approved by the local institutional review board.

### Study Design

Cardiovascular risk factors including: tobacco smoking, hypercholesterolemia, hypertension and family history of cardiovascular disease diagnosed in first-degree relatives before 55 years of age were assessed. Baseline demographic data and cardiovascular medications were recorded in all subjects [7]. Hypertension was defined as either resting systolic or diastolic blood pressure >140 or >90mmHg, at two different clinical visits or the prescription of anti-hypertensive medications. Hypercholesterolemia was defined as fasting total plasma cholesterol of ≥4.9mmol/L or the prescription of lipid-lowering medications. Smoking status was recorded as ever-smoker (past or current) or non-smoker.

Anthropometric measurements including body weight and waist-hip circumference ratio (WHR) were performed. Body-mass index (BMI) was calculated as kg/m^2^. Systolic and diastolic blood pressures were measured. Fasting blood samples were obtained to measure serum levels of glucose, HbA1c, total cholesterol, triglyceride, low-density lipoprotein-cholesterol (LDL-C), high-density lipoprotein-cholesterol (HDL-C), creatinine and number of circulating EPCs. Glomerular filtration rate (GFR) was calculated using the Modification of Diet in Renal Disease Study equation.

#### Arterial stiffness

Arterial stiffness was measured non-invasively, by a single experienced operator, with the VP-2000 System (Colin Corp., USA) and represented as brachial to ankle pulse wave velocity (baPWV). Previous studies have validated that baPWV was closely correlated with aortic PWV as a measurement of arterial stiffness [8]. Patients were allowed to rest for 5 minutes and the sites of maximum arterial pulsation were determined by physical examination. Sequential recordings of pressure waveforms at the brachial and posterior tibial arteries were made using hand-held manometer probes with simultaneous electrocardiogram gating. Measurements were taken after achieving coherent reproduction of signals with maximum amplitudes.

Transmission time (T_BA_) was defined as the time interval between the initial increase in brachial and posterior tibial waveforms. Transmission distance from the suprasternal notch to the brachium (D_B_) and ankle (D_A_) were determined by direct superficial measurement. The value baPWV was automatically calculated as the transmission distance divided by the transmission time: baPWV = (D_A _- D_B_)/T_BA_. After obtaining bilateral baPWV values, the mean value of the two was used for subsequent analysis. Intra-observer variability testing revealed an intra-class correlation coefficient of 0.89 *(P < 0.001*).

#### Circulating endothelial progenitor cells

Circulating EPCs were measured by fluorescence-activated cell analysis of a peripheral blood sample and defined by the expression of surface markers: CD34/KDR^+ ^and CD133/KDR^+ ^[7]. In brief, 100 μl of peripheral blood was incubated with a phycoerythrin-conjugated monoclonal antibody against human KDR (Sigma, St Louis, Missouri, USA), followed by a fluorescein isothiocyanate (FITC)-conjugated CD34 and CD133 antibodies (Beckman Coulter, Fullerton, California, USA). FITC-labeled anti-human CD45 antibody was used for differential gating during flow analysis. FITC-labeled IgG1a (Beckman Coulter) and phycoerythrin-labeled IgG2b (Becton Dickinson, Franklin Lakes, New Jersey, USA) served as the isotypic control for color compensation. Analysis was performed with an automated fluorescence-activated cell counter (Elite; Beckman Coulter) in which 1,000,000 events were counted. The percentages of all the measured components defined as the absolute cell counts divided by the lymphocyte counts were calculated. Intra-observer variability testing revealed an intra-class correlation coefficient of 0.90 *(P < 0.001)*.

### Statistical analysis

Data were expressed as mean ± standard deviation for continuous variables and proportions for categorical variables. Baseline characteristics were compared between groups using independent samples *t*-test or Fisher's exact test, as appropriate. Patients with type 2 DM were divided into those with satisfactory glycemic control (HbA1c < 6.5%) and those with poor glycemic control (HbA1c ≥6.5%).

Since the distribution pattern of baPWV and the number of circulating EPCs were highly skewed, these variables were log-transformed to normalize their distribution before analysis. Correlation coefficients were calculated to assess the association between the circulating EPCs and baPWV. Stepwise backward linear regression analysis was used to identify independent predictors for circulating EPCs and baPWV. Only parameters with *P < 0.1 *in uni-variate analysis were subsequently entered into a multi-variate model. All statistical analyses were performed using the statistical package SPSS for Windows (Version 15.0, SPSS, Chicago, USA). A *P *value *< 0.05 *was considered as statistically significant.

## Results

### Clinical characteristics

As shown in Table 1, there were no significant differences in age or sex between patients with type 2 DM and controls (all *P>0.05*). Patients with type 2 DM had significantly higher prevalence of hypertension, hypercholesterolemia and smokers, greater BMI and WHR, and more likely to be prescribed with anti-hypertensive medications, statins and aspirin (Table 1, *P < 0.01*). Furthermore, the serum fasting glucose, HbA1c, triglyceride and creatinine levels were higher and the GFR was lower in DM patients as compared with controls (Table 1, *P < 0.01*). In contrast, DM patients had significantly lower serum total cholesterol and LDL-C than controls which were likely due to an increased use of statins in DM patients.

**Table 1:** Clinical characteristics of patients with DM and Controls

	DM patients n = 234	Controls n = 121	*P*-value
Age, years	56.5 ± 7.6	56.5 ± 8.4	0.99
Males, n (%)	119 (51)	59 (49)	0.74
Hypertension, n (%)	96 (41)	18 (15)	< 0.01
Hypercholesterolemia, n (%)	114 (49)	24 (20)	< 0.01
Ever-smokers, n (%)	72 (31)	7 (6)	< 0.01
Duration of DM, yrs	10.0 ± 8.0	-	
Body-mass index, kg/m^2^	25.1 ± 4.7	21.5 ± 3.2	< 0.01
Waist-hip circumference ratio	0.9 ± 0.1	0.8 ± 0.1	< 0.01
Body weight, kg	64.6 ± 10.6	56.4 ± 9.1	< 0.01
Systolic blood pressure, mmHg	144.2 ± 21.2	118.8 ± 22.9	< 0.01
Diastolic blood pressure, mmHg	79.6 ± 8.8	72.3 ± 8.2	< 0.01
Fasting blood glucose, mmol/L	7.2 ± 2.2	4.9 ± 0.5	< 0.01
HbA1c, %	7.7 ± 1.5	5.8 ± 0.3	< 0.01
Triglyceride, mmol/L	1.6 ± 1.2	1.3 ± 0.9	< 0.01
Total cholesterol, mmol/L	4.6 ± 0.9	5.1 ± 0.8	< 0.01
LDL-C, mmol/L	2.6 ± 0.8	2.9 ± 0.7	< 0.01
HDL-C, mmol/L	1.3 ± 0.4	1.6 ± 0.4	< 0.01
Creatinine, μmol/L	84.6 ± 27.2	71.0 ± 16.6	< 0.01
GFR, ml/min/1.73m^2^	55.4 ± 15.3	60.0 ± 13.7	< 0.01
Beta-blocker, n (%)	106 (45%)	10 (8%)	< 0.01
Calcium channel blocker, n (%)	77 (33%)	3 (2%)	< 0.01
ACEI/ARB, n (%)	122 (52%)	6 (5%)	< 0.01
Aspirin, n (%)	103 (44%)	9 (7%)	< 0.01
Statin, n (%)	119 (51%)	9 (7%)	< 0.01
Insulin, n (%)	35 (15%)	0 (0%)	< 0.01
Log CD34/KDR^+ ^EPCs, 10^-3^/ml	0.96 ± 0.31	1.23 ± 0.36	< 0.01
Log CD133/KDR^+ ^EPCs, 10^-3^/ml	0.70 ± 0.26	1.13 ± 0.38	< 0.01
Log baPWV, cm/s	3.24 ± 0.08	3.18 ± 0.09	< 0.01

**Abbreviations: **T2DM = Type 2 diabetes mellitus; HbA1c = hemoglobin A1c; LDL-C = low-density lipoprotein cholesterol; HDL-C = high-density lipoprotein cholesterol; GFR = glomerular filtration rate; ACEI = angiotensin converting enzyme inhibitor; ARB = angiotensin receptor blocker; EPC = endothelial progenitor cell; baPWV = brachial-ankle pulse wave velocity

Among those patients with DM, 120/234 (51%) of them had satisfactory glycemic control with HbA1c < 6.5%. As expected, DM patients with satisfactory glycemic control had a significantly lower serum fasting glucose and HbA1c levels compared with those with poor glycemic control (Table 2, *P < 0.01*). Furthermore, DM patients with satisfactory glycemic control had a lower prevalence of hypertension, hyperlipidemia, smoking history and were less frequently prescribed with insulin than those with poor glycemic control (Table 2, *P < 0.05*). However, there were no significant differences in the serum lipid profile, GFR and use of cardiovascular medications between the two groups (Table 2, *P>0.05*).

**Table 2:** Clinical characteristics of DM patients with satisfactory (HbA1c < 6.5%) or poor (HbA1c≥6.5%) glycemic control

	HbA1c < 6.5% (n = 120)	HbA1c≥6.5% (n = 114)	*P*-value
Age, years	56.5 ± 8.5	56.8 ± 8.4	0.81
Males, n (%)	62 (52)	57 (50)	0.35
Hypertension, n (%)	40 (33)	58 (51)	< 0.05
Hypercholesterolemia, n (%)	52 (43)	62 (54)	< 0.01
Ever-smokers, n (%)	29 (24)	43 (38)	< 0.01
Duration of DM, yrs	9.7 ± 7.0	10.3 ± 8.6	0.37
Body-mass index, kg/m^2^	25.4 ± 4.3	24.9 ± 5.2	0.48
Waist-hip circumference ratio	0.9 ± 0.1	0.9 ± 0.1	0.38
Body weight, kg	64.2 ± 11.7	65.6 ± 10.3	0.52
Systolic blood pressure, mmHg	145.2 ± 23.2	143.1 ± 18.5	0.45
Diastolic blood pressure, mmHg	77.9 ± 7.8	80.4 ± 9.5	0.08
Fasting blood glucose, mmol/L	5.7 ± 1.2	8.5 ± 2.3	< 0.01
HbA1c, %	6.1 ± 0.5	8.8 ± 1.3	< 0.01
Triglyceride, mmol/L	1.5 ± 1.1	1.7 ± 1.3	0.18
Total cholesterol, mmol/L	4.6 ± 0.9	4.5 ± 0.9	0.27
LDL-C, mmol/L	2.6 ± 0.8	2.5 ± 0.7	0.21
HDL-C, mmol/L	1.3 ± 0.4	1.2 ± 0.4	0.06
Creatinine, μmol/L	82.8 ± 21.6	85.7 ± 31.0	0.42
GFR, ml/min/1.73m^2^	56.4 ± 12.1	55.0 ± 17.2	0.39
Beta-blocker, n (%)	56 (47)	50 (44)	0.34
Calcium channel blocker, n (%)	35 (29)	42 (37)	0.08
ACEI/ARB, n (%)	57 (48)	65 (57)	0.06
Aspirin, n (%)	48 (40)	55 (48)	0.13
Statin, n (%)	57 (48)	62 (54)	0.11
Insulin, n(%)	9 (8)	26 (23)	< 0.01
Log CD34/KDR^+ ^EPCs, 10^-3^/ml	1.05 ± 0.27	0.88 ± 0.32	< 0.01
Log CD133/KDR^+ ^EPCs, 10^-3^/ml	0.78 ± 0.28	0.61 ± 0.21	< 0.01
Log baPWV, cm/s	3.23 ± 0.08	3.27 ± 0.09	< 0.05

Abbreviations as in Table 1

### Circulating endothelial progenitor cells and arterial stiffness

Compared with controls, patients with type 2 DM had significantly lower circulating Log CD34/KDR^+ ^EPCs (0.96 ± 0.31 10^-3^/ml vs. 1.23 ± 0.36 10^-3^/ml, *P < 0.01*) and Log CD133/KDR^+ ^EPCs (0.70 ± 0.26 10^-3^/ml vs. 1.13 ± 0.38 10^-3^/ml, *P < 0.01*). Among patients with DM, those with satisfactory glycemic control had significantly higher levels of circulating Log CD34/KDR^+ ^EPCs (1.05 ± 0.27 10^-3^/ml vs. 0.88 ± 0.32 10^-3^/ml, *P < 0.01*) and Log CD133/KDR^+ ^EPCs (0.78 ± 0.28 10^-3^/ml vs. 0.61 ± 0.21 10^-3^/ml, *P < 0.01*) than those with poor glycemic control.

Arterial stiffness as measured by Log baPWV, was significantly greater in patients with type 2 DM as compared with controls (3.24 ± 0.08 cm/s vs. 3.18 ± 0.09 cm/s, *P < 0.01*). Among patients with DM, those with satisfactory glycemic control had significantly lower Log baPWV than those with poor glycemic control (3.23 ± 0.08 cm/s vs. 3.27 ± 0.09 cm/s, *P < 0.05*).

**Figure 1:**
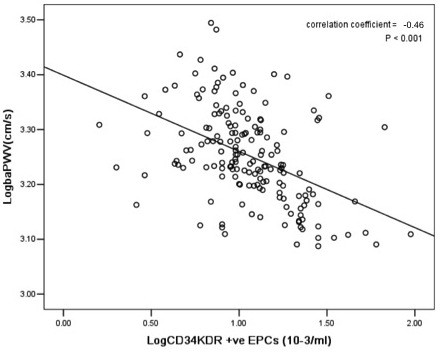
**Relationship between levels of circulating Log CD34/KDR^+ ^EPCs with Log baPWV in patients with DM**.

**Figure 2:**
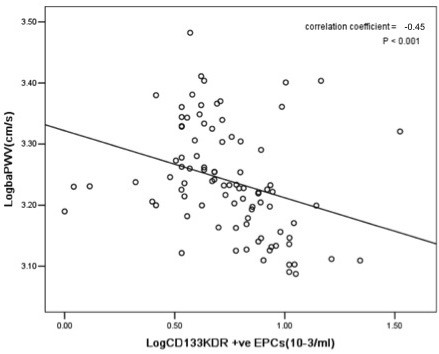
**Relationship between levels of circulating Log CD133/KDR^+ ^EPCs with Log baPWV in patients with DM**.

**Figure 3:**
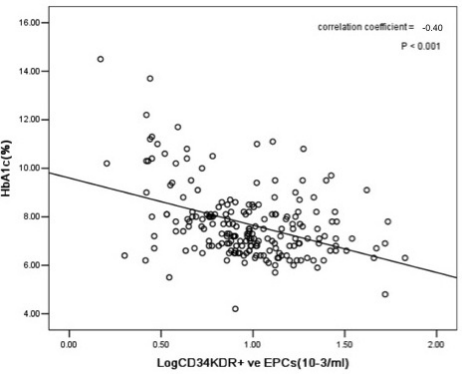
**Relationship between levels of circulating Log CD34/KDR^+ ^EPCs and HbA1c in patients with DM**.

**Figure 4:**
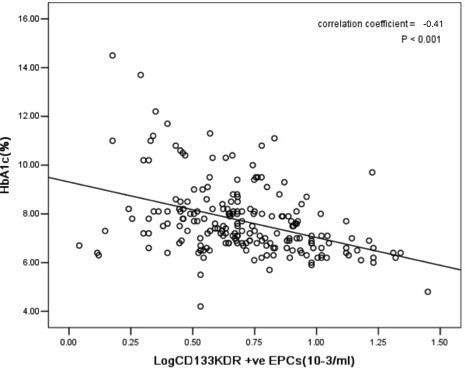
**Relationship between levels of circulating Log CD133/KDR^+ ^EPCs and HbA1c in patients with DM**.

The circulating levels of Log CD34/KDR+ EPCs (r = -0.46, *P < 0.001*, Figure 1) and Log CD133/KDR+ EPCs (r = -0.45, *P < 0.001*, Figure 2) were negatively correlated with Log baPWV. Whilst the level of HbA1c positively correlated with Log baPWV (r = 0.20, *P < 0.05*) and negatively correlated with circulating levels of Log CD34/KDR+ EPCs (r = -0.40, *P < 0.001*, Figure 3) and Log CD133/KDR+ EPCs (r = -0.41, *P < 0.001*, Figure 4).

Multivariate regression analysis revealed that in patients with diabetes, HbA1c, diabetes duration, Log CD34/KDR^+ ^EPCs and Log CD133/KDR^+ ^EPCs were independent predictors of Log baPWV (Table 3).

**Table 3:** Clinical predictors for arterial stiffness in patients with DM

	Uni-variate		Multi-variate	
	B (95% Confidence Interval)	*P*-value	B (95% Confidence Interval)	*P*-value
Ever-smokers, n (%)	0.011 (-0.002, 0.024)	0.20		
Duration of diabetes, yrs	0.002 (0.001, 0.003)	0.09	0.002 (0.001, 0.002)	< 0.05
Systolic blood pressure, mmHg	0.001 (0.000, 0.004)	0.30		
HbA1c, %	0.013 (0.005, 0.021)	< 0.05	0.010 (0.005, 0.014)	< 0.01
Total cholesterol, mmol/L	0.012 (0.002, 0.026)	< 0.05	0.014 (0.006, 0.021)	0.17
Creatinine, μmol/L	0.000 (0.000, 0.001)	0.66		
Log CD34/KDR^+ ^EPCs, 10^-3^/ml	-0.096 (-0.197, -0.005)	< 0.05	-0.021 (-0.032, -0.003)	< 0.01
Log CD133/KDR^+ ^EPCs, 10^-3^/ml	-0.144 (-0.224, -0.064)	< 0.05	-0.024 (-0.044, -0.003)	< 0.01

Abbreviations as in Table 1

## Discussion

Our results demonstrate a close inter-relationship between glycemic control, circulating EPCs and arterial stiffness in patients with type 2 DM. Patients with type 2 DM had a lower level of circulating EPCs and increased arterial stiffness compared with normal controls. Amongst those with diabetes, a poor glycemic control correlated with a lower number of EPCs as well as increased arterial stiffness, whilst better glycemic control correlated with a relatively greater number of EPCs as well as a lesser degree of arterial stiffness. Furthermore, after adjusting for other cardiovascular risk factors and medications, glycemic control and EPC counts were identified as independent risk predictors for arterial stiffness.

The mechanisms by which hyperglycemia leads to an increase in arterial stiffness remain unclear but is likely related to an imbalance between the protective versus detrimental pathways. Vascular endothelial cells secrete large amounts of chemical mediators and biologically active substances that actively regulate vascular tone and permeability [9]. Among those protective substances secreted, NO is one of the main factor which has vasodilatory, anti-platelet, anti-inflammatory and anti-oxidant properties for maintenance of endothelial function [10]. However, in the presence of chronic hyperglycemia, polyol, protein kinase C and pentose phosphate pathways are enhanced. These pathways are detrimental to the vascular endothelium and results in an increased oxidative stress and accelerated endothelial cell apoptosis [11,12]. Furthermore, the availability and biological activity of NO are also reduced in hyperglycemic states, resulting in worsening endothelial dysfunction [13-16]. In addition, hyperglycemia induces vascular smooth muscle cell proliferation as well as promotes their conversion from the contractile phenotype to the synthentic phenotype, thus promoting the development of arterial stiffness [17].

On the other hand, circulating EPCs may play an important role in repairing damaged vascular endothelium [18]. Prior studies have shown that the number of circulating EPCs was positively correlated to endothelial function [19]. However, in hyperglycemic states, EPCs exhibit impaired proliferation, adhesion, and incorporation into vascular structures [20]. An impairment of vascular NO system has also been shown to result in the dysfunction and depletion of EPCs [4]. Indeed, the levels of circulating EPCs were reduced in patients with type 2 DM as compared with normal subjects, and have been shown to have a significant inverse correlation with serum glucose and HbA1c levels [21]. EPCs have also been found to be reduced in pre-diabetic states (impaired fasting glucose and impaired glucose tolerance) with further significant reductions in number at the clinical onset of diabetes and after ~20 years of disease [22]. Moreover, recent interesting studies noted reduced numbers and functions of EPCs in subjects who are predisposed to having DM (e.g. polycystic ovarian syndrome), indicating that reduced EPCs may even be a risk factor of DM [23].

Consistent with previous studies [24,25], the present study confirmed that patients with DM have a blunted endothelium regenerating capacity as reflected by a decrease in number of circulating EPCs and was associated with large artery atherosclerosis as measured by PWV. Importantly, our results further demonstrated that the degree of hyperglycemia control in those patients with type 2 DM was closely related to the levels of circulating EPCs as well as the arterial stiffness. In this study, DM patients who could achieve satisfactory hyperglycemia had significantly higher circulating level of EPCs and lower arterial stiffness. This might be one of the mechanisms in which satisfactory glycemic control in type 2 DM patients (HbA1c < 6.5%) had reduced cardiovascular events [26]. On the other hand, recent studies have also shown that an over vigorous lowering of HbA1c to < 6.0% has been associated with increased all-cause mortality [27]. The mechanism of such observations has yet to be determined but is less likely to be related to a change in PWV and EPC number according to our findings.

This study has several limitations. First, this was only a modest size cross-sectional study of approximately 350 subjects and the result needs further confirmation in other cohorts. Second, the causal relationship between reduced circulating EPC and arterial stiffness could not be confirmed. Whether improved glycemic control in DM patients can increase circulating EPCs and decrease arterial stiffness needs to be confirmed in future prospective studies. Third, the effect of hyperinsulinemia and insulin resistance on circulating EPCs counts, endothelial function and arterial stiffness were not assessed in the present study. Forth, in addition to the number of circulating EPCs, the function of EPCs might also play an important role in vascular repair. Unfortunately, the function of EPCs was not measured in this study.

## Conflicts of interests

None of the authors has a real or perceived conflict of interest or has received any personal or financial support.

## Authors' contributions

WSY planned the study, collected the data, performed the statistical analysis and was involved in the preliminary writing of the manuscript. KKL and CWS was involved in writing of the manuscript. MW, GHY and KHY was involved in data collection. HFT supervised the project and with writing of the manuscript. All authors read and approved the final manuscript.
